# Supplementary material for: A digital intervention to improve mental health and interpersonal resilience in young people who have experienced technology‐assisted sexual abuse: a feasibility clinical trial
Source: Child Adolesc Ment Health. 2026 Jan 6;31(1):23–32. doi: 10.1111/camh.70060 (PMC12832212; doi:10.1111/camh.70060)
Supplement: Supplementary file 1 — Figure S1. Screenshots of the i‐Minds app. Figure S2. Standardised deprivation index scores. Figure S3. Bar charts illustrating self‐reported frequency of exposure to potentially harmful online materials and interactions in the previous year (Items 1–4). Figure S4. Bar charts illustrating self‐reported frequency of exposure to potentially harmful online materials and interactions in the previous year (Items 5–8). Figure S5. Consort diagram illustrating participant flow through the i‐Minds trial. Figure S6. Bar charts illustrating the additional subjective feedback provided as part of the uMARS. Figure S7. Histograms for impact of i‐Minds app on mental health (Items 1–4). Figure S8. Histograms for impact of the i‐Minds app on mental health (Items 5 and 6). Table S7. Summary of open feedback of uMARS items. Table S1. Baseline characteristics and service use. Table S2. Standardised deprivation index scores. Table S3. Breakdown of retention by recruitment site. Table S4. Intervention accessed via own phone or study handset. Table S5. Descriptive statistics for the general app satisfaction items. Table S6. Descriptive statistics for the uMARS. Table S8. Perceived impact of using the i‐Minds app on mental health understanding. Table S9. Mental health outcome data at baseline and follow‐up assessments. Table S10. Reliable change index analysis across clinical outcomes. [file CAMH-31-23-s001.docx]

**A digital intervention to improve mental health and interpersonal resilience in young people who have experienced Technology-Assisted Sexual Abuse: a feasibility clinical trial**

**Supporting Information**

**Figure S1.** Screenshots of the i-Minds app

 
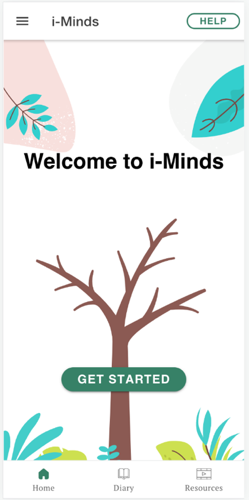

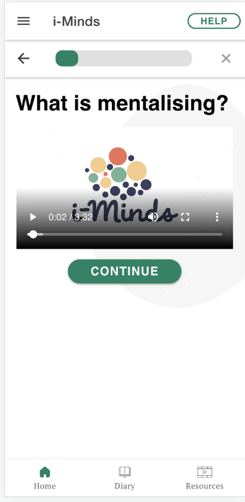

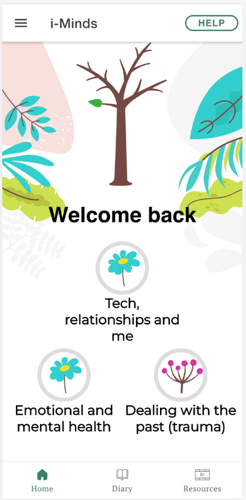


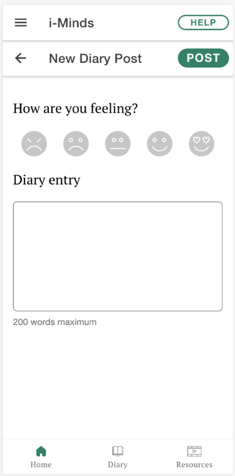

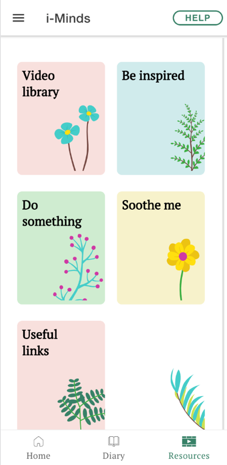

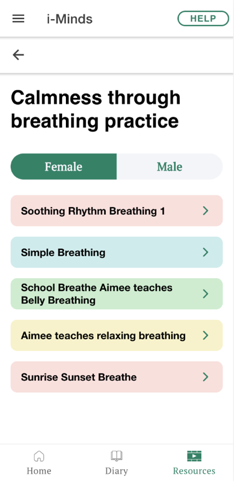


**Table S1.** Baseline Characteristics and service use

| Characteristic | | N (%) |
| --- | --- | --- |
| Age (years) | Mean (min-max) | 15.42 (12.03 – 18.06) |
| Gender | Man / Male (n/%) | 6 (13.9%) |
|  | Woman / Female (n/%) | 30 (69.8%) |
|  | Non-binary / Third gender | 4 (9.3%) |
|  | I would rather not say / unsure | 3 (7.0%) |
| Gender matches sex assigned at birth | Yes | 33 (76.7%) |
|  | No | 9 (20.9%) |
|  | Prefer not to say | 1 (2.3%) |
| Ethnicity | White British | 43 (95.3%) |
|  | Asian/Asian British | 1 (2.3%) |
|  | Black British | 1 (2.3%) |
| Deprivation Index | Range | Scotland: 278 – 6,933  England: 4,282 – 32,503 |
| Baseline service use and use of internet / being online | | N (%) |
| Currently supported by CAMHS | No | 2 (4.4%) |
|  | CAMHS / Child & Adolescent Mental Health Team | 34 (75.6%) |
|  | Sexual Assault Referral Centre (SARC) | 3 (6.7%) |
|  | e-therapy provider | 3 (6.7%) |
| Length of time receiving help from services (months) | [N] Mean (SD) min-max | 16.09 (22.89) 1 - 96 |

**Table S2.** Standardised deprivation index scores

| Decile | Frequency | % |
| --- | --- | --- |
| 1 | 5 | 11.6 |
| 2 | 4 | 9.3 |
| 3 | 5 | 11.6 |
| 4 | 4 | 9.3 |
| 5 | 3 | 7.0 |
| 6 | 5 | 11.6 |
| 7 | 4 | 9.3 |
| 8 | 5 | 11.6 |
| 9 | 4 | 9.3 |
| 10 | 3 | 7.0 |
| Total | 42 | 97.7 |

*Each decile represents approximately 10% of England/Scotland's population from the most deprived 10% of the population to the least deprived 10%. Data zones in decile1 fall within the most deprived 10% of areas nationally, and data zones in decile 10 fall within the least deprived 10% of zones nationally.

**Figure S2.** Standardised deprivation index scores


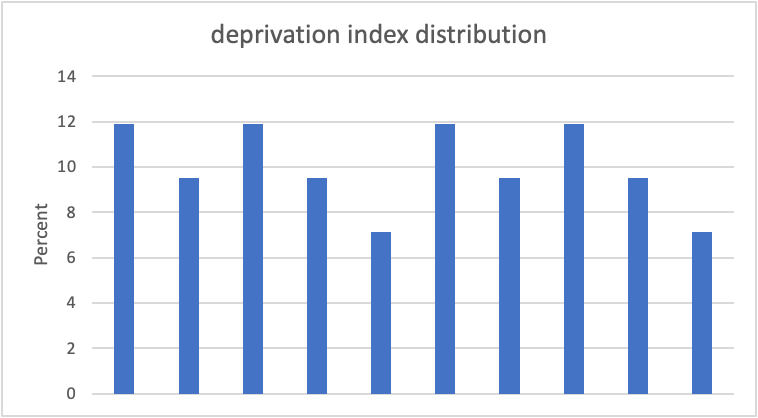


1 2 3 4 5 6 7 8 9 10

4

Deprivation Decile (1 = Most Deprived, 10 = Least Deprived)

**Figure S3.** Bar charts illustrating self-reported frequency of exposure to potentially harmful online materials and interactions in the previous year (Items 1-4)
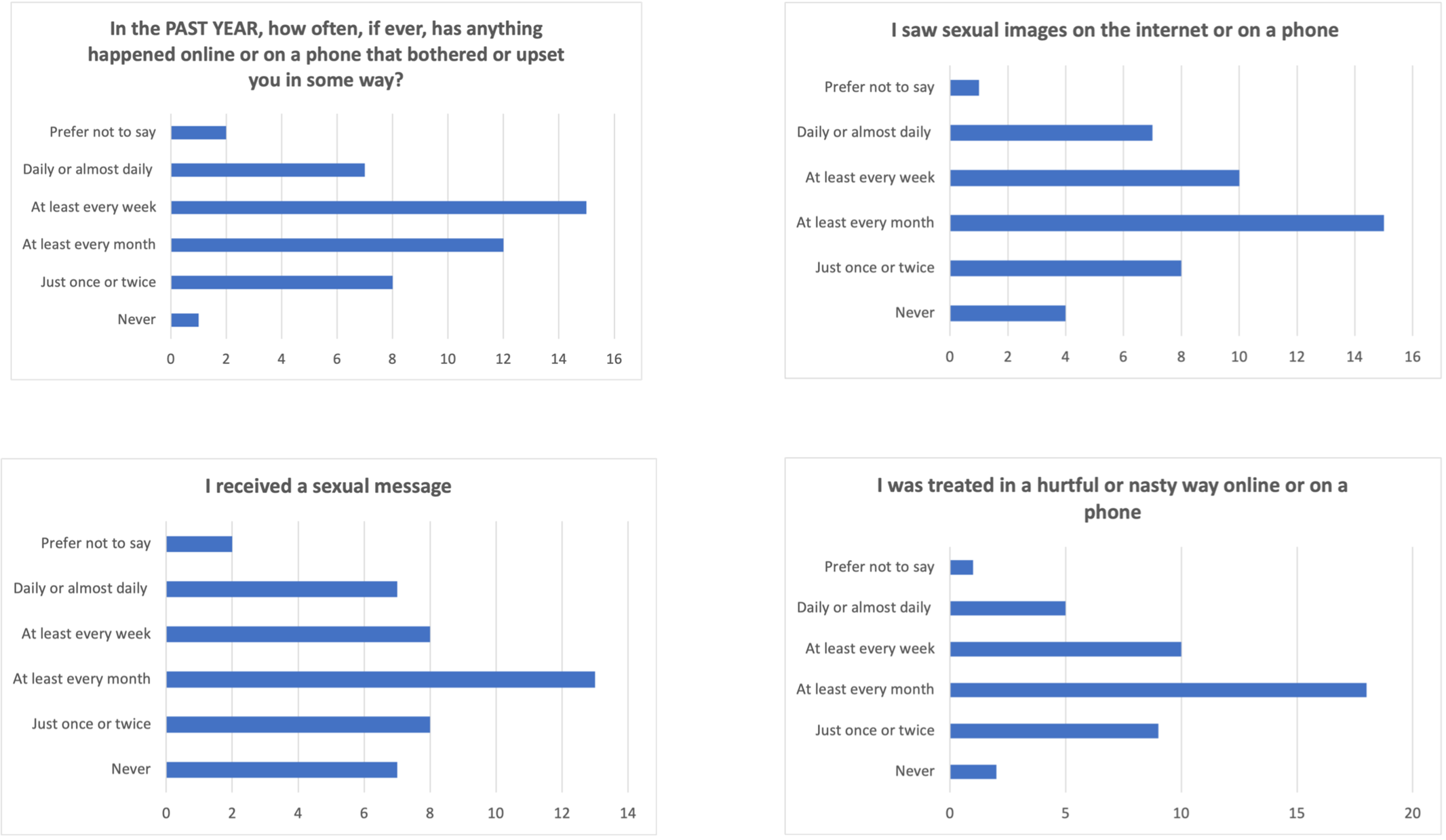


Percentage of participants

Percentage of participants

Percentage of participants

Percentage of participants

**Figure S4.** Bar charts illustrating self-reported frequency of exposure to potentially harmful online materials and interactions in the previous year (Items 5-8)

**
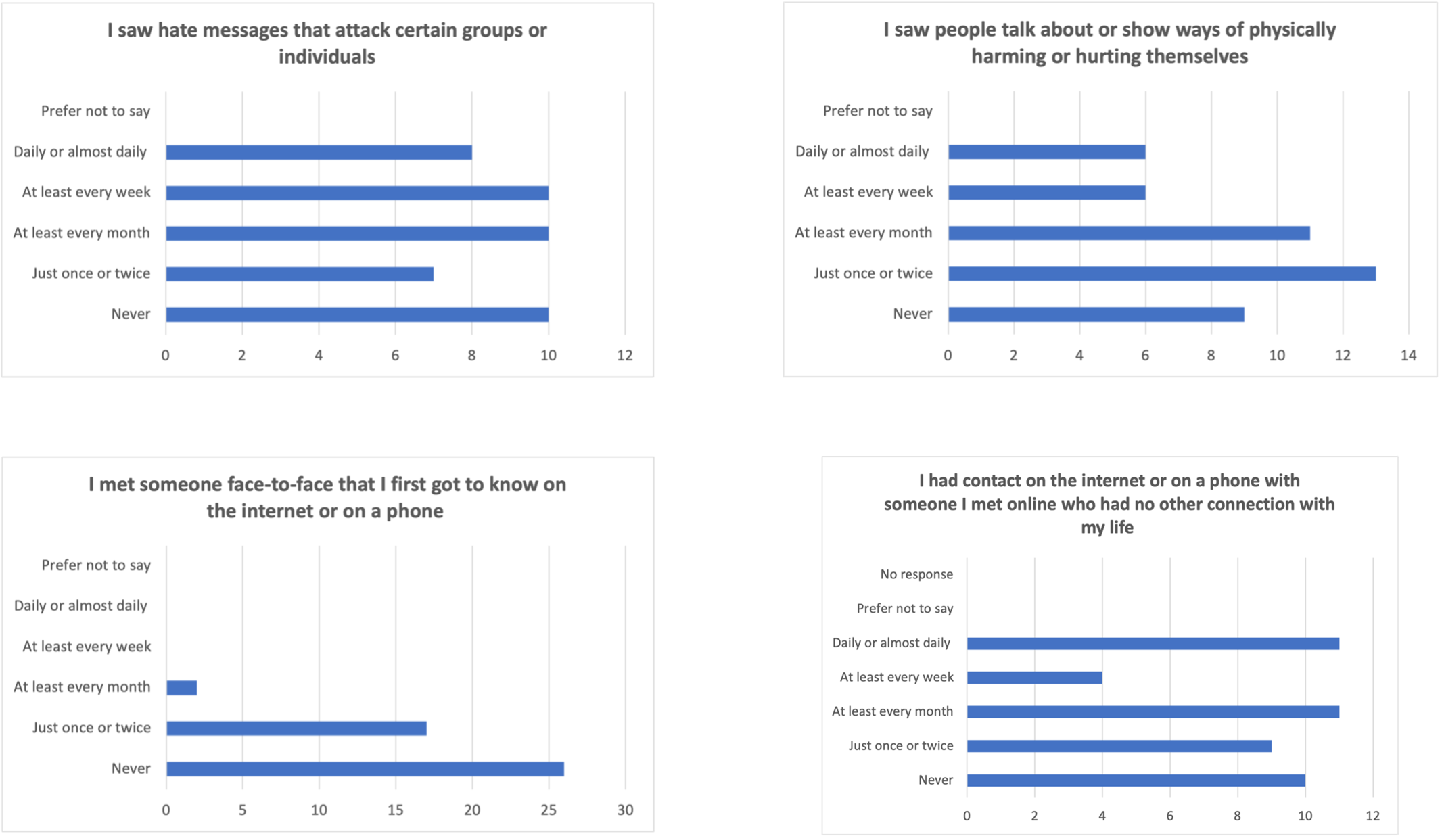
**

Percentage of participants

Percentage of participants

Percentage of participants

Percentage of participants

**Figure S5.**  Consort diagram illustrating participant flow through the i-Minds trial

Screened by e-therapy service for eligibility (n= 74)

Assessed for eligibility (referred to trial) (n= 72)

Excluded (n= 29)

- Not meeting inclusion criteria (n= 8)

i) not aged 12 to 18 years (n = 2)

iii) not receiving support from NHS CAMHS, SARC or e-therapy providers (Kooth)and/or not continuing to be actively supported by the service over the duration of the trial (n = 3);

iii) are at risk of current or recent (past month) suicidality (n = 3)

- Declined to participate (n= 12)
- Unable to contact (n = 6)
- Withdrawal during baseline (n = 3)

Lost to follow-up (give reasons) (n= 6)

- Moved out of area (n =1)
- Discharged from service (n =1)
- Unable to contact (n=4)

Received intervention (n= 43)

Completed follow up assessments: (n = 37)

Allocation

Assessment

Follow-Up

Completed baseline assessment (n= 43)

Enrollment

Screened by NHS service for eligibility (n= 73)

Excluded (n= 4)

- Declined to participate (n = 2)
- Discontinued engagement with service (n = 2)

Screened

**Table S3.** Breakdown of retention by recruitment site

| **Site** | **Baseline completed** | **Follow-up completed** | **% retention** |
| --- | --- | --- | --- |
| **Edinburgh** | 28 | 23 | 82.1.% |
| **Manchester** | 13 | 12 | 92.3% |
| **e-therapy provider** | 2 | 2 | 100.0% |
| **Total** | 43 | 37 | 86.0% |

**Table S4.** Intervention accessed via own phone or study handset

|  | **Edinburgh** | **Manchester** | **e-therapy provider** | **Total** |
| --- | --- | --- | --- | --- |
| **Own Phone** | 25 | 12 | 2 | 39 |
| **Study Handset** | 3 | 1 | 0 | 4 |
| **Handset returned post-intervention** | 2 | 1 | 0 | 3 |
| **Handset not returned post-intervention (reason)** | 1 (taken by police as evidence in an ongoing investigation) | 0 | 0 | 1 |

**Table S5.** Descriptive statistics for the general app satisfaction items

| Item* | N | Mean (SD) | Min-Max | Median |
| --- | --- | --- | --- | --- |
| 1. Did using the app take a lot of work? | 37 | 2.16 (1.57) | 1-7 | 2 |
| 2. Did using the app take up a lot of time? | 37 | 2.49 (1.30) | 1-7 | 3 |
| 3. Was it difficult to keep the/your Smartphone with you or carry it around? | 37 | 1.32 (1.27) | 1-7 | 1 |
| 4. Did you ever lose or forget the Smartphone? | 37 | 1.81 (1.76) | 1-6 | 1 |
| 5. Do you think other young people would find the app easy to use? | 37 | 5.78 (1.47) | 2-7 | 6 |
| 6. Overall, was using the i-Minds app Stressful | 37 | 2.03 (1.66) | 1-6 | 1 |
| 7. Overall, was using the i-Minds app challenging | 37 | 2.24 (1.59) | 1-7 | 2 |
| 8. Overall, was using the i-Minds app enjoyable? | 37 | 4.73 (1.54) | 1-7 | 5 |
| 9. Did using the i-Minds app make you feel worse? | 37 | 1.89 (1.35) | 1-6 | 1 |
| 10. Did using the i-Minds app make you feel better? | 37 | 4.59 (1.72) | 1-7 | 5 |
| 11. Do you think it would be helpful for your keyworker/GP to see how you answered the questions in the app? | 37 | 3.38 (1.98) | 1-7 | 4 |

**All items rated on a 7-point Likert scale (1 = Not at all; 7 = Very much so)*

**Table S6.** Descriptive statistics for the uMARS

|  | N | Mean (SD) | Min. | Max. | Median | Range of possible scores |
| --- | --- | --- | --- | --- | --- | --- |
| Subscale / Item | | | | | | |
| Engagement | 2 | 17.06 (3.62) |  | 23 | 17.50 | 5-25 |
| 1. Entertainment | 36 | 3.33 (0.82) | 1 | 5 | 3 |  |
| 1. Interest | 36 | 3.89 (0.95) |  | 5 | 4 |  |
| 1. Customisation | 36 | 2.35 (1.31) | 1 | 5 | 2 |  |
| 1. Interactivity | 35 | 3.58 (1.34) | 1 | 5 | 4 |  |
| 1. Target Group | 36 | 4.39 (0.72) | 3 | 5 | 5 |  |
| Functionality | 36 | 17.78 (1.90) | 12 | 20 | 18 | 4-20 |
| 1. Performance | 36 | 4.46 (0.80) | 2 | 5 | 5 |  |
| 1. Ease of use | 36 | 4.43 (0.76) | 2 | 5 | 5 |  |
| 1. Navigation | 36 | 4.33 (0.63) | 3 | 5 | 4 |  |
| 1. Gestural Design | 36 | 4.57 (0.65) | 3 | 5 | 5 |  |
| Aesthetics | 36 | 12.83 (1.48) | 9 | 15 | 13 | 3-15 |
| 1. Layout | 36 | 4.54 (0.65) | 3 | 5 | 5 |  |
| 1. Graphics | 36 | 4.16 (0.69) | 3 | 5 | 4 |  |
| 1. Visual Appeal | 36 | 4.08 (0.83) | 2 | 5 | 4 |  |
| Information | 36 | 15.97 (2.86) | 6 | 20 | 16.50 | 4-20 |
| 1. Quality of Information | 36 | 4.51 (0.77) | 2 | 5 | 5 |  |
| 1. Quantity of Information | 36 | 4.19 (1.10) | 1 | 5 | 5 |  |
| 1. Visual Information | 26 | 3.93 (1.03) |  | 5 | 4 |  |
| 1. Credibility of Source | 35 | 4.50 (0.61) | 3 | 5 | 5 |  |

**Figure S6.** Bar charts illustrating the additional subjective feedback provided as part of the uMARS

**
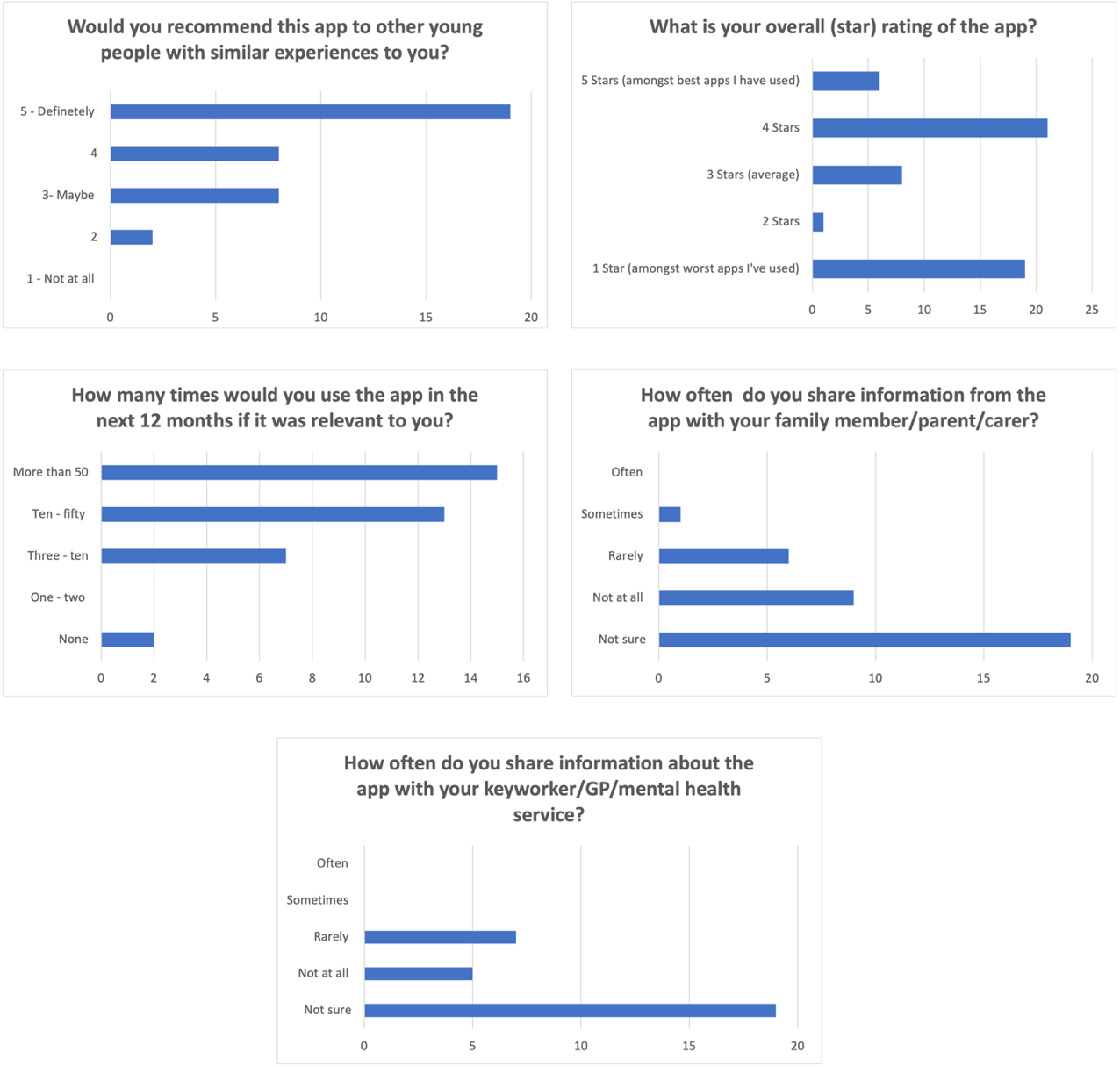
**

Percentage of participants

Percentage of participants

Percentage of participants

Percentage of participants

Percentage of participants

**Table S7.** Summary of open feedback of uMARS items

| **uMars domain** | **Open feedback** |
| --- | --- |
| **Engagement** | Users liked the animation style of the app |
|  | Include more fun, engaging material |
|  | Include gamification features (e.g., word search exercise) |
|  | Include more customisation features (e.g., tree motif presented in different colours/backgrounds; more choice of background/text colour) |
|  | Diary consistently reported as the most helpful aspect of the app |
| **Functionality** | Navigation features of the app require improvement |
|  | Include speech-to-text functionality to overcome text-heavy nature of the app |
| **Aesthetics** | Liked the look-and-feel of the app metaphor and imagery (e.g. progress bars; tree motif flourishing with leaves as participant’s worked through the app) |
|  | Add more customisable features |
| **Information** | The content of the app reflected users’ difficulties and experiences |
|  | Inviting users to put themselves “in another’s shoes” (i.e., to encourage mentalisation) helped users process personal experiences more objectively |
|  | Videos: soe users said the videos helpfully explained the content of the app; others felt the videos require improvement (e.g., clearer explanations, more accessible length) |
|  | Less “wordy”/text heavy |
|  | Include more trigger warnings in the app |

**Table S8.** Perceived impact of using the i-Minds app on mental health understanding

|  |  | **Mean (SD)** | **Min.** | **Max.** | **Median** | **Range of possible scores** |
| --- | --- | --- | --- | --- | --- | --- |
| ***Using the i****-****Minds app…*** | | | | | | |
| … increased my awareness on the importance of my mental health | 36 | 3.40 (0.96) | 1 | 5 | 3 | 1-5 |
| …. increased my knowledge/understanding of my mental health | 36 | 3.51 (0.93) | 1 | 5 | 4 | 1-5 |
| … has changed my attitudes towards improving my mental health | 36 | 3.13 (1.18) | 1 | 5 | 3 | 1-5 |
| … has made me more motivated to address my mental health | 36 | 3.40 (1.12) | 1 | 5 | 4 | 1-5 |
| … would encourage me to seek further help for my mental health (if I needed it) | 35 | 3.59 (1.12) | 1 | 5 | 4 | 1-5 |
| … will make me do things/activities that will help my mental health | 36 | 3.21 (1.18) | 1 | 5 | 3 | 1-5 |

**Figure S7.** Histograms for impact of i-Minds app on mental health (Items 1-4)


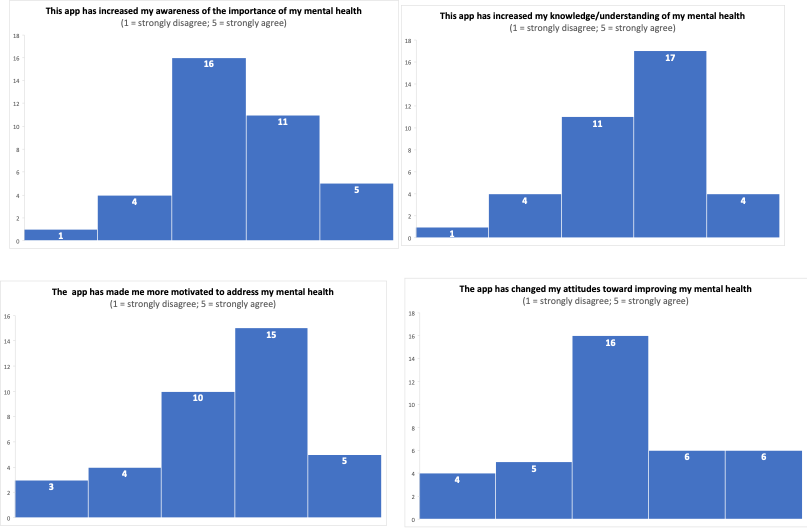


5-point Likert scale

1. 5

5-point Likert scale

1. 5

5-point Likert scale

1. 5

5-point Likert scale

1. 5

**Figure S8.** Histograms for impact of the i-Minds app on mental health (Items 5 and 6)

**
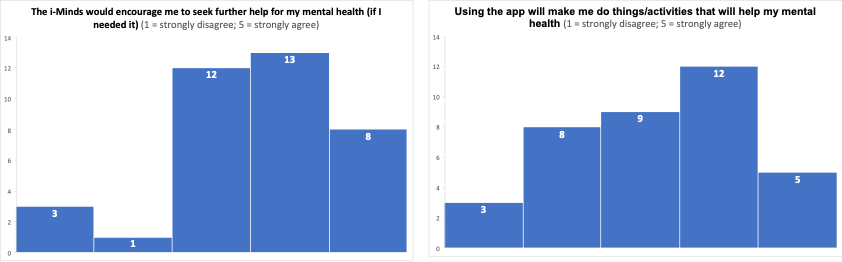
**

**Table S9.** Mental health outcome data at baseline and follow-up assessments

|  |  | Baseline | | | |  | Follow-up | | | Follow-up – Baseline | | |
| --- | --- | --- | --- | --- | --- | --- | --- | --- | --- | --- | --- | --- |
| Scale | **Scale** | **N** | **%**  **missing** | **Mean (SD)** | **Range** | **N** | **%**  **missing** | **Mean (SD)** | **Range** | **N** | **Mean (SD)** | **95% CI** |
| RFQ | Certainty | 42 | 2.32% | 1.74 (2.52) | 0 – 9 | 34 | 20.93% | 1.97(2.81) | 0-10 | 34 | 0.29(3.22) | [-0.83,1.42] |
|  | Uncertainty | 42 | 2.32% | 10.81 (3.51) | 1 – 17 | 34 | 20.93% | 9.50(3.70) | 2-18 | 34 | -1.12(3.76) | [-2.43,0.19] |
| PRIUSS | **Total** | 37 | 13.95% | 48.19 (15.97) | 19 – 87 | Not administered at follow-up | | | | | | |
|  | Social impairment | 39 | 9.30% | 15.46(5.38) | 6-27 |  |  |  |  |  |  |  |
|  | Emotional impairment | 39 | 9.30% | 13.15(5.56) | 5-25 |  |  |  |  |  |  |  |
|  | Risk/impulsive | 37 | 13.95% | 19.57(7.12) | 7-35 |  |  |  |  |  |  |  |
| RCADS-25 | **Total** | 40 | 6.98% | 43.30(17.13) | 8 – 73 | 34 | 20.93% | 38.71 (16.02) | 10 - 73 | 33 | -3.70(10.49) | [-7.42,0.02] |
|  | Social phobia | 41 | 4.65% | 6.51(2.38) | 1-9 | 34 | 20.93% | 6.06(2.68) | 1-9 | 34 | -0.44(1.83) | [-1.08,0.20] |
|  | Panic disorder | 40 | 6.98% | 4.80(2.89) | 0-9 | 34 | 20.93% | 4.71(2.52) | 0-9 | 33 | -0.09(1.97) | [-0.79,0.61] |
|  | Major depression | 41 | 4.65% | 18.83(7.72) | 3-30 | 34 | 20.93% | 16.18(6.58) | 1-29 | 34 | -1.88(5.16) | [-3.68,-0.08] |
|  | Separation anxiety | 42 | 2.32% | 3.79(2.41) | 0-9 | 34 | 20.93% | 3.18(2.35) | 0-9 | 34 | -0.44(1.67) | [-1.02,0.14] |
|  | Generalised anxiety | 41 | 4.65% | 5.59(2.65) | 0-9 | 34 | 20.93% | 4.94(2.50) | 1-9 | 34 | -0.56(2.15) | [-1.31,0.19] |
|  | Obsessive compulsive | 41 | 4.65% | 3.49(3.17) | 0-9 | 34 | 20.93% | 3.65(2.81) | 0-9 | 34 | 0.24(2.63) | [-0.68,1.15] |
| CRIES | **Total** | 43 | 0.00% | 48.65 (10.51) | 16 - 63 | 36 | 16.28% | 44.78 (13.01) | 11 - 65 | 36 | -3.89(11.48) | [-7.77,-0.00] |
|  | Intrusion | 43 | 0.00% | 9.98(3.84) | 0-15 | 37 | 13.95% | 8.81(4.47) | 0-15 | 37 | -1.32(3.38) | [-3.62,-0.03] |
|  | Avoidance | 43 | 0.00% | 16.00(3.84) | 5-20 | 37 | 13.95% | 14.86(4.21) | 3-20 | 37 | -0.95(4.19) | [-2.34,0.45] |
|  | Arousal | 43 | 0.00% | 19.02(4.96) | 7-25 | 36 | 16.28% | 17.80(6.08) | 5-25 | 36 | -1.03(5.72) | [-2.96,0.91] |
| DERS-SF | **Total** | 41 | 4.65% | 63.05 (14.04) | 36 – 85 | 34 | 20.93% | 61.18 (14.26) | 34 – 90 | 33 | -0.70(11.17) | [-4.66,3.26] |
|  | Awareness | 42 | 2.32% | 9.43(3.28) | 3-15 | 34 | 20.93% | 9.35(3.58) | 3-15 | 34 | 0.03(2.95) | [-1.00,1.06] |
|  | Clarity | 42 | 2.32% | 9.31(3.45) | 3-15 | 34 | 20.93% | 9.26(3.21) | 4-15 | 34 | -0.18(3.24) | [-1.31,0.95] |
|  | Goals | 42 | 2.32% | 10.93(2.74) | 3-15 | 34 | 20.93% | 10.71(2.84) | 6-15 | 34 | -0.21(2.68) | [-1.14,0.73] |
|  | Impulse | 41 | 4.65% | 10.78(3.38) | 3-15 | 34 | 20.93% | 10.06(3.09) | 5-15 | 33 | -0.30(2.73) | [-1.27,0.67] |
|  | Non-Acceptance | 42 | 2.32% | 11.17(2.65) | 3-15 | 34 | 20.93% | 10.62(2.89) | 5-15 | 34 | -0.35(2.67) | [-1.29,0.58] |
|  | Strategy | 42 | 2.32% | 11.43(2.91) | 6-15 | 34 | 20.93% | 11.18(2.79) | 6-15 | 34 | 0.03(1.83) | [-0.61,0.67] |
| ISM | **Total** | 38 | 11.62% | 109.3 (14.1) | 63–129 | 32 | 25.58% | 108.4(14.6) | 80-132 | 31 | -1.29(15.0) | [-6.79, 4.21] |
|  | Awareness | 40 | 6.98% | 23.78(4.25) | 10-25 | 34 | 20.93% | 23.06(3.81) | 14-28 | 34 | -0.71(7.35) | [-2.02, 0.61] |
|  | Approval | 39 | 9.30% | 25.33(3.56) | 16-31 | 32 | 25.58% | 25.88(3.50) | 18-30 | 32 | 0.28(83.52) | [-0.99,-1.55] |
|  | Separation anxiety | 40 | 6.98% | 25.80(4.75) | 11-32 | 34 | 20.93% | 24.56(4.83) | 14-32 | 34 | -0.97(4.73) | [-2.62,-0.61] |
|  | Timidity | 40 | 6.98% | 21.00 (4.31) | 11-30 | 34 | 20.93% | 21.11(3.68) | 15-29 | 34 | 0.06(4.42) | [-1.48, 1.60] |
|  | Fragile | 39 | 9.30% | 14.05(3.62) | 5-20 | 34 | 20.93% | 14.32(3.29) | 7-20 | 34 | 0.00(3.57) | [-1.27,1.27] |
| ECR-RC | **Total** | 38 | 11.62% | 47.71 (19.34) | 12 - 83 |  | Not administered at follow-up | | | | | |
| CD-RISC-10 | **Total** | 40 | 6.98% | 15.58 (7.33) | 3 – 36 | 34 | 20.93% | 18.12 (7.87) | 4 – 35 | 34 | 2.24(6.24) | [0.06,4.41] |

***Notes:*** *RFQ-Y – Reflective Functioning Questionnaire for Youth – higher scores indicate greater reflective functioning; PRIUSS - Problematic and Risky Internet Use Screening Scale – higher scores indicate greater levels of problematic internet use; RCADS -25 – Revised Children’s Anxiety and Depression Scale 25 item version – higher scores indicate more severe anxiety and depression symptoms; CRIES - Child Revised Impact of Events Scale – higher scores indicate higher PTSD symptoms; DERS-SF – Difficulties in Emotion Regulation Short Form – higher scores indicate greater difficulty with emotion regulation; ISM – Interpersonal Sensitivity Measure – higher scores indicate higher levels of interpersonal difficulty; ECR-RC - Experiences in Close Relationships Scale - Revised Child version – higher scores indicate greater levels of attachment difficulty; CD-RISC-10 – Connor-Davidson Resilience Scale 10 item – higher scores indicate higher resilience.*

**Table S10.** Reliable change index analysis across clinical outcomes

| Measure | Sample Size  (n) | No Change  n (%) | Improve  n (%) | Deteriorate  n (%) |
| --- | --- | --- | --- | --- |
| RFQ-C | 34 | 29 (85.3%) | 4 (11.8%) | 1 (2.9%) |
| RFQ-U | 34 | 20 (58.8%) | 10 (29.4%) | 4 (11.8%) |
| CRIES | 36 | 26 (72.2%) | 7 (19.4%) | 3 (8.3%) |
| DERS-SF | 33 | 24 (72.7%) | 5 (15.2%) | 4 (12.1%) |
| ISM | 31 | 21 (67.7%) | 5 (16.1%) | 5 (16.1%) |
| CD-RISC | 34 | 28 (82.4%) | 6 (17.6%) | 0 (0.0%) |

***Notes:*** *CRIES - Child Revised Impact of Events Scale; DERS-SF – Difficulties in Emotion Regulation Short Form; ISM – Interpersonal Sensitivity Measure; CD-RISC-10 – Connor-Davidson Resilience Scale 10 item; RFQ-C, Reflective Functioning Questionnaire for Youth-Certainty; RFQ-U, Reflective Functioning Questionnaire for Youth-Uncertainty.*

Reliable Change Index (RCI) analyses were performed using the Excel calculator and analytic procedures provided by Morsley & Dowzer (2014). Benchmarking data for relevant control / normative samples of participants in a similar age range were extracted from published reports which utilised the above research measures. More specifically, the following sources were used:

For the RFQ (both RFQ-C and RFQ-U): Quattropani, M. C., Geraci, A., Lenzo, V., Sardella, A., & Schimmenti, A. (2022). Failures in reflective functioning, dissociative experiences, and eating disorder: A study on a sample of Italian adolescents. *Journal of Child & Adolescent Trauma*, *15*(2), 365-374.

For CRIES:   Verlinden, E., van Meijel, E. P., Opmeer, B. C., Beer, R., de Roos, C., Bicanic, I. A., Lamers-Winkelman, F., Olff, M., Boer, F., & Lindauer, R. J. (2014). Characteristics of the Children's Revised Impact of Event Scale in a clinically referred Dutch sample. *Journal of traumatic stress*, *27*(3), 338–344. <https://doi.org/10.1002/jts.21910>

For the DERS:   Lydon-Staley, D. M., Xia, M., Mak, H. W., & Fosco, G. M. (2019). Adolescent Emotion Network Dynamics in Daily Life and Implications for Depression. *Journal of abnormal child psychology*, *47*(4), 717–729. https://doi.org/10.1007/s10802-018-0474-y

For ISM: Bhutani, R., Sudhir, P. M., & Philip, M. (2014). Teasing experiences, interpersonal sensitivity, self-schema and psychological distress in youth: an exploratory study. *Psychological Studies*, *59*(3), 241-251, <https://doi.org/10.1007/s12646-014-0261-y>

For the CD-RISC-10: She, R., Yang, X., Lau, M. M. C., & Lau, J. T. F. (2020). Psychometric properties and normative data of the 10-item Connor-Davidson Resilience Scale among Chinese adolescent students in Hong Kong. *Child psychiatry and human development*, *51*(6), 925–933. https://doi.org/10.1007/s10578-020-00970-1
